# Supplementary material for: Disrupted brain structural connectivity in Pediatric Bipolar Disorder with psychosis
Source: Sci Rep. 2019 Sep 20;9:13638. doi: 10.1038/s41598-019-50093-4 (PMC6754428; doi:10.1038/s41598-019-50093-4)
Supplement: Supplementary file 1 — Supplementary Information [file 41598_2019_50093_MOESM1_ESM.pdf]

Title: Disrupted brain structural connectivity in Pediatric Bipolar Disorder with psychosis

Authors: Henrique M. Fernandes, Joana Cabral, Tim J. Van Hartevelt, Louis-David Lord, Carsten Gleesborg, Arne Moller, Gustavo Deco, Peter C. Whybrow, Predrag Petrovic, Anthony C. James, and Morten L. Kringelbach

Supplementary Information

Graph Theory Metrics

The following graph theoretical metrics were computed in the present study.

Connection Density. Connection density, or wiring cost, is the actual number of edges in the graph as a proportion of the total number of possible edges. For an undirected graph with N nodes without self-connections, the total number of possible connections *is* $(N * (N - 1))/2$.

Degree. The degree of a node *d(n)* is calculated as the number of nodes to which node *n* is connected.

Characteristic Path Length. The shortest path length between areas *n* and *p* is calculated as the average shortest path length in the network, i.e. the minimum number of connections, or minimum cost needed to connect regions *n* and *p*, where a connection cost, or edge length, is the inverse of connection weight. The characteristic path length is given as the mean shortest path length over all pairs of nodes in the network, calculated as the global mean of the distance matrix.

Global Efficiency. Global efficiency, E_{global}, reflects how efficiently information can be exchanged over the network and is defined by the mean of the inverse shortest path length, L_{ij}, between each pair of nodes in a network, C, with N nodes (52):

$$E_{global}(C) = \frac{1}{N(N-1)} \sum_{i \neq j \in C} \frac{1}{L_{ij}}$$

Nodal Efficiency. The nodal efficiency, $E_{nodal}(i)$, reflects how well a node connects to all other nodes in the network and is defined as the mean of the inverse shortest path length, L_{ij}, between a node, *i,* and all other nodes in the network:

$$E_{nodal}(i) = \frac{1}{N-1} \sum_{i \neq j \in C} \frac{1}{d_{ij}}$$

Local Efficiency. The local efficiency of a network, E_{local}, is defined as the average *Nodal Efficiency*, and indicates globally how information is transferred within the neighbours of a given node.

$$E_{local}(C) = \frac{1}{N} \sum_{i \in C} E_{nodal}(i)$$

Clustering coefficient. The clustering coefficient reflects the extent of local interconnectivity in a network by considering the fraction of a node's neighbours that are also neighbours of each other. It is measured as the fraction of connected triangles, δ_v, to the total number of triples in the network, τ_v. A network's weighted clustering coefficient is then defined as the average of the clustering coefficient over all nodes.

$$Cl(C) = \frac{1}{|V'|} \sum_{v \in V'} \frac{\delta_v}{\tau_v}$$

where V' is the subset of nodes with degree >2.

Small-worldness. A network C is considered to be small-world (σ>1), if the average shortest path length, L, is small and the weighted clustering coefficient, Cl, is high, when compared to equivalent random networks (L_R and Cl_R, respectively) (53).

$$\sigma\,(C) = \frac{Cl/Cl_R}{L/L_R}$$

In this study we generated 100 matched random networks, preserving the degree distribution and ensuring connectedness. The ratio between the original and the generated random networks, for both the weighted clustering coefficient and weighted path lengths measures, allows the correction for network differences across individuals with regards to edge number and degree distribution.

Group consistency in structural connectivity

To evaluate the on the sensitivity of connectivity estimations (and further graph estimates) to individual differences in data quality, we analysed the consistency of group signatures of SC, i.e. how consistent are the patterns of estimated SC across all subjects in a group, as well as between groups. To achieve this, we used the following two strategies for evaluating group consistency (GC) in SC:

GC-I Intra-group consistency measured as the correlation (Pearson's) between each subject's SC (upper diagonal) and group mean SC. The resulting *r* values where *z*-transfomed (Fisher-Z transformation), before averaging, and converting (inverse of Fisher-Z) the resultant group consistency average back to *r* scale. This value represents the within-group consistency, i.e. for each group, how well all subject's SC correlate with the group's average SC.

GC-II Intra-group consistency measured as the distribution (Pearson's) of correlations between all possible pairs of subjects in a group. The resulting distribution of all pairwise (pairs of subjects) SC comparisons are represented as a histogram. This indicates how well SCs in a group correlate with each other. Inter-group consistency was also assessed by considering all subjects as part of the same group.

Supplementary Figures

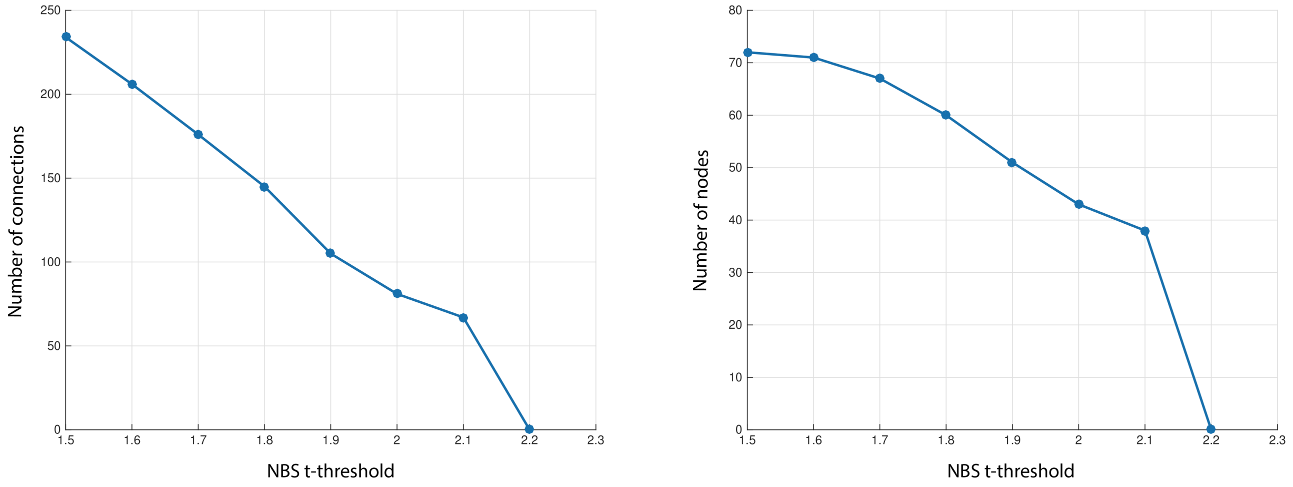

Supp. Figure 1. Network-based statistics. Relationship between t-threshold and number of connections/nodes, for the largest anatomical component. The t-threshold used in this study (*2.1*) was selected based on the maximal t-threshold ≥ 2.0 where a component was found, and generated an NBS component with approximately 43% nodes of the network and 71links.

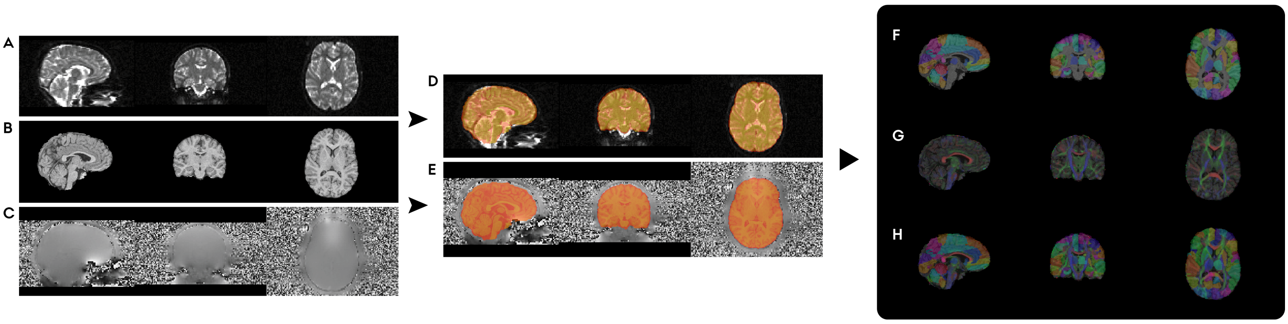

Supp. Figure 2. Example of a subject's complete dataset and the corresponding outputs from multiple preprocessing steps. A) DTI sequence, represented here with the first B0 volume. **B)** Skull-stripped T1 scan. **C)** Fieldmap shows the spatial distribution of potential sources of signal dropout or eddy-current induced distortions. **D)** T1 overlaid on the diffusion volume (with transparencyset to 50% – α=0.5), shows a very high level of spatial correspondence between these two types of images. **E)** T1 overlaid on the fieldmap, reveals potential effects of signal dropout are very contained, and mainly constricted to a small portion of the OFC. **F)** AAL atlas overlaid on the T1, reveal a very good fits of the standard anatomical labelling of brain regions with the subject's individual brain. **G)** Results from diffusion tensor fitting after correction for head motion and eddy-currents) to our diffusion dataset overlaid on the T1 (V1 modulated by FA at α=0.5), are indicative of the quality of our diffusion dataset, with main tracts across the brain well defined and color-coded according to their direction (e.g. red: corpus-callosum; green: parts of the inferior fronto-occipital tract; blue: cortico-spinal tract). **H)** Superimposed F) and G) show the fit between the anatomical atlas (α=0.5) and the diffusion data (α=0.5; overlaid on the T1).

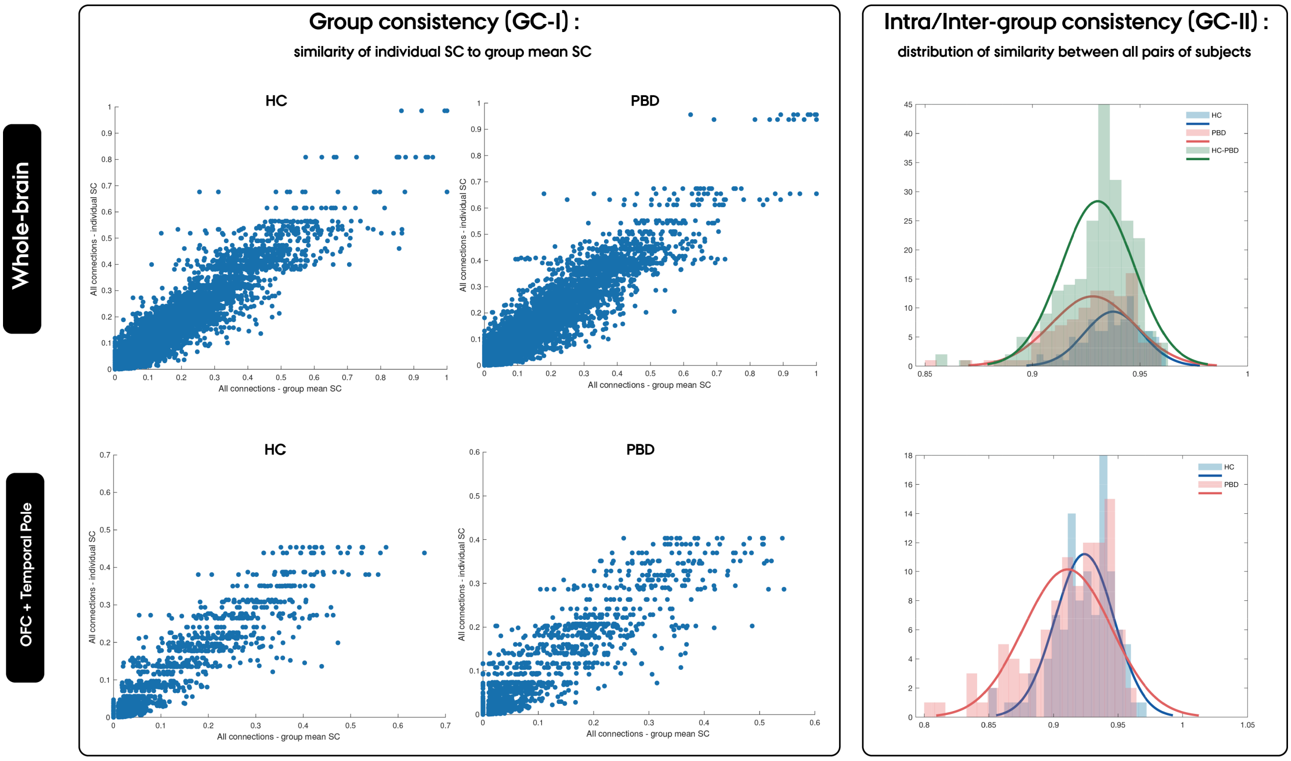

Supp. Figure 3. Consistent group signatures of SC for HC and PBD patients with psychosis. The left panel, intra-group consistency is estimated based *measured as the association between individual SC signatures and group average SC. and found that the two groups reveal a very high level of intra-group consistency, for the whole-brain (top; HC: 97,1%; PBD: 96,7%) as well as a for a subnetwork comprising regions of the OFC and temporal pole (bottom; HC: 96,5%; PBD: 96,0%), which are susceptible to signal dropout in diffusion sequences. The right panel shows the degree of association between the signatures of SC for all pairs of subjects in the same group. Again, it is well evident the high level of SC group consistency, for the whole-brain (top; 100% and 94% of all pairwise combinations in the HC and PBD groups respectively have a correlation higher than r=0.85, with number of occurrence peaking at approximately r=0.94, for both populations) as well as for the OFC-Temporal Pole subnetwork (both groups peaking at approximately 94% consistency level). The overlap between the distributions of intra-group consistency of both groups is further confirmed by the inter-group consistency distribution (HC-PBD: peak at ~ 93%). Together, these results indicates that, at both global and local (OFC and Temporal Pole regions) levels, the patterns of SC are highly consistent between groups. This strongly suggests that potential differences in data quality (due to e.g. head-motion and eddy-currents), are not disrupting the robustness of the estimation of the SC patterns (highly consistency within and between groups).*

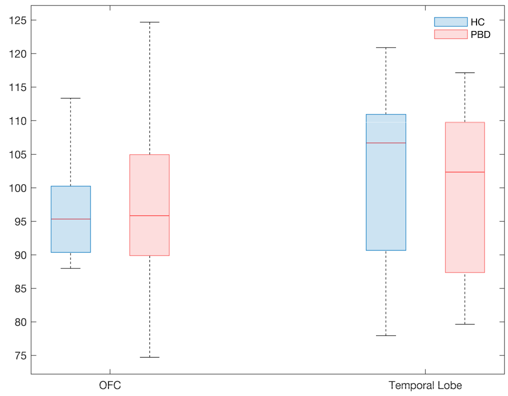

Supp. Figure 4. Mean signal of the OFC and temporal pole regions in the diffusion images, for HC and PBD patiets with psychosis. Brain regions affected by signal dropout are characterised by increased heterogeneity in the signal across region voxels (gradient-like effect along the boundary between e.g. sinuses and the OFC). The mean signal (of all non-zero voxels) in the first b0 volume, for the OFC and temporal pole AAL masks was calculated to assess potential group differences in data quality in these regions. Our results clearly indicate that there are no trends of difference between groups (OFC: p=0.87; Temporal Pole: p=0,50).

Supplementary Tables

Supp. Table 1. Connections comprising the connected component of significant structural connectivity difference between PBD and HC ($p < 0.05$).

Area1			Area2		Difference
name	n		name	n	
TempGPol_s (R)	42	<->	Amyg (R)	84	-214.01
Amyg (L)	39	<->	PaHippG (L)	41	-204.23
TempGPol_m (R)	42	<->	Amyg (R)	88	-194.00
TempG_s (R)	80	<->	HeschlG (R)	82	-192.49
OrbFG_m (R)	6	<->	OrbF_s (R)	26	-123.43
Pallid (L)	27	<->	Rectus (L)	75	-119.22
Caud (R)	14	<->	TriFG_i (R)	72	-119.15
Caud (R)	28	<->	Rectus (R)	72	-103.65
TempGPol_s (R)	74	<->	Putam (R)	84	-77.86
HeschlG (R)	74	<->	Putam (R)	80	-74.36
Cing_p (L)	34	<->	Cing_m (R)	35	-67.60
Cing_m (R)	35	<->	Cing_p (L)	34	-67.60
Olf (L)	6	<->	OrbF_s (R)	21	-65.92
Cing_a (R)	6	<->	OrbF_s (R)	32	-64.35
PaCLob (L)	1	<->	PreCG (L)	69	-58.63
Cing_a (L)	6	<->	OrbF_s (R)	31	-58.11
FG_s (L)	1	<->	PreCG (L)	3	-57.25
TempG_m (L)	37	<->	Hipp (L)	85	-48.71
Thalam (L)	11	<->	OpeFG_i (L)	77	-47.34
Caud (L)	6	<->	OrbF_s (R)	71	-38.73
Insula (R)	22	<->	Olf (R)	30	-38.08
HeschlG (R)	52	<->	Occ_m (R)	80	-36.55
Pallid (R)	28	<->	Rectus (R)	76	-33.81
FG_s_m (R)	8	<->	FG_m (R)	24	-26.14
Insula (L)	25	<->	OrbFG_m (L)	29	-25.07
FG_s_m (R)	6	<->	OrbF_s (R)	24	-24.98
TempG_m (R)	48	<->	LingG (R)	86	-22.83
Cing_a (R)	14	<->	TriFG_i (R)	32	-22.72
FG_m (R)	3	<->	FG_s (L)	8	-22.63
TempG_s (R)	52	<->	Occ_m (R)	82	-22.02
Caud (R)	2	<->	PreCG (R)	72	-22.00
Caud (R)	7	<->	FG_m (L)	72	-21.47
Thalam (L)	29	<->	Insula (L)	77	-20.05
TempGPol_s (R)	80	<->	HeschlG (R)	84	-17.98
Caud (L)	11	<->	OpeFG_i (L)	71	-12.95
Cing_m (R)	14	<->	TriFG_i (R)	34	-11.26

Cing_m (R)	1	<->	PreCG (L)	34	-9.94
RolOpe (L)	3	<->	FG_s (L)	17	-9.79
Thalam (L)	34	<->	Cing_m (R)	77	-9.29
TempG_i (L)	81	<->	TempG_s (L)	89	12.83
Occ_s (R)	43	<->	Calcar (L)	50	14.42
Thalam (L)	33	<->	Cing_m (L)	77	17.51
TempG_m (L)	29	<->	Insula (L)	85	18.62
Hipp (R)	4	<->	FG_s (R)	38	21.31
SuMargG (R)	30	<->	Insula (R)	64	24.93
Hipp (R)	10	<->	OrbFG_m (R)	38	26.24
PaHippG (L)	27	<->	Rectus (L)	39	29.38
Precun (L)	39	<->	PaHippG (L)	67	36.22
Cing_a (L)	19	<->	SuppMoA (L)	31	36.54
TempG_m (R)	84	<->	TempGPol_s (R)	86	39.53
Calcar (R)	30	<->	Insula (R)	44	41.23
Cing_a (L)	3	<->	FG_s (L)	31	41.93
TempG_i (R)	84	<->	TempGPol_s (R)	90	45.74
Precun (R)	40	<->	PaHippG (R)	68	51.65
Hipp (R)	22	<->	Olf (R)	38	52.88
Caud (L)	41	<->	Amyg (L)	71	53.27
PaHippG (R)	6	<->	OrbF_s (R)	40	59.97
PaHippG (R)	16	<->	OrbFG_i (R)	40	75.49
Amyg (R)	16	<->	OrbFG_i (R)	42	76.48
Calcar (R)	43	<->	Calcar (L)	44	89.78
Calcar (L)	44	<->	Calcar (R)	43	89.78
TempG_m (L)	81	<->	TempG_s (L)	85	91.46
TempG_m (L)	83	<->	TempGPol_s (L)	85	98.73
TempG_i (R)	86	<->	TempG_m (R)	90	103.68
TempG_i (R)	88	<->	TempGPol_m (R)	90	119.24
Pallid (R)	4	<->	FG_s (R)	76	127.12
FusifG (R)	38	<->	Hipp (R)	56	129.35
Cing_a (L)	23	<->	FG_s_m (L)	31	129.51
Amyg (L)	21	<->	Olf (L)	41	161.91
PaHippG (R)	38	<->	Hipp (R)	40	280.90
Pallid (R)	74	<->	Putam (R)	76	325.58

Supp. Table 2. Hubs of the brain for the PBD and HC groups, according to three different classification methods.

Hubs					
Efficiency		Connector Hubs		Provincial Hubs	
**PBD**	HC	PBD	HC	PBD	HC
L Rolandic Oper	L Rolandic Oper	L Hippocampus	L Hippocampus	L Caudate	L Caudate
R Rolandic Oper	R Rolandic Oper	L Precuneus	**R Hippocampus**	R Caudate	R Caudate
L Occipital Sup	**R Front Med Orb**	L Putamen	L Precuneus	L Pallidum	L Pallidum
L Fusiform	**L Cuneus**	R Cingulum Post	L Putamen	R Pallidum	L Pallidum
R Fusiform	L Occipital Sup	**R Occipital Sup**	**R Putamen**	L Cingulum Post	L Cingulum Post
L Parietal Inf	**L Occipital Inf**		R Cingulum Post	L ParaHippocamp	L ParaHippocamp
R Parietal Inf	L Fusiform		**R Amygdala**	R ParaHippocamp	R ParaHippocamp
R Heschl	R Fusiform			L Fusiform	L Fusiform
**R Temp Pole m**	L Parietal Inf			R Fusiform	R Fusiform
	R Parietal Inf			R Parietal Inf	R Parietal Inf
	R Heschl			**R Putamen**	**L Frontal Sup Orb**
	**L Temp Inf**			**R Insula**	**R Frontal Sup Orb**
	**R Temp Inf**				**L Postcentral**
					**R Heschl**

Supp. Table 3. Partial Correlation coefficients between nodes with significant group difference in E_{nodal} and neurocognitive/psychotic symptoms, for both populations.

		Partial Correlation Coefficient					
Network metric	Group	FIQ	Coding	P4Ex	P5Gr	N1BA	N2EW
E_{nodal} of ' L Front Sup Orb '	HC	0,06	-	-	-	-	-
	BPD	0,36	0,41	0,48	0,31	-0,27	-0,41
E_{nodal} of ' L Front Mid Orb '	HC	0,03	-	-	-	-	-
	BPD	-0,21	-0,21	-0,17	-0,30	0,38	0,57 *
E_{nodal} of ' L Front Inf Orb '	HC	0,36	-	-	-	-	-
	BPD	0,77 *	0,15	0,05	0,18	-0,24	-0,38
E_{nodal} of ' R Rolandic Oper '	HC	0,18	-	-	-	-	-
	BPD	0,27	0,52	-0,09	-0,23	0,10	0,05
E_{nodal} of ' R Front Med Orb '	HC	0,42	-	-	-	-	-
	BPD	0,14	0,04	-0,01	-0,06	0,10	-0,29
E_{nodal} of ' R Insula '	HC	-0,07	-	-	-	-	-
	BPD	0,31	0,40	-0,07	-0,19	-0,02	0,00
E_{nodal} of ' L Occipital Inf '	HC	0,07	-	-	-	-	-
	BPD	0,00	0,16	-0,12	-0,03	0,19	0,26
E_{nodal} of ' R SupraMarginal '	HC	0,20	-	-	-	-	-
	BPD	-0,11	0,11	-0,16	-0,11	0,23	-0,23
E_{nodal} of ' R Temporal Inf '	HC	-0,08	-	-	-	-	-
	BPD	0,40	0,17	-0,19	-0,38	0,09	-0,10
E_{nodal} of ' R Cingulum Mid '	HC	0,25	-	-	-	-	-
	BPD	0,36	0,45	0,05	0,01	-0,06	-0,37
E_{nodal} of ' R Heschl '	HC	0,26	-	-	-	-	-
	BPD	-0,53	-0,12	0,32	0,25	0,03	0,00
E_{nodal} of ' R Temporal Sup '	HC	-0,34	-	-	-	-	-
	BPD	-0,01	0,32	-0,03	0,17	-0,10	-0,38

- $p < 0.05$

Supp. Table 4. Partial Correlation coefficients between global graph theory metrics and neurocognitive/mood scores, for both populations.

		Partial Correlation Coefficient					
Network metric	Group	FIQ	Coding	P4Ex	P5Gr	N1BA	N2EW
Mean Connectivity *[1]	HC	0,04	-	-	-	-	-
	PBD	0,29	0,32	-0,20	0,02	-0,27	-0,19
Total Number of Fibers	HC	0,29	-	-	-	-	-
	PBD	0,40	0,34	0,10	-0,01	0,05	-0,37
Fibers per Connection	HC	0,37	-	-	-	-	-
	PBD	0,40	0,26	0,11	0,19	-0,15	-0,35
Degree	HC	0,05	-	-	-	-	-
	PBD	0,28	0,30	0,07	-0,13	0,13	-0,28
Connection density	HC	0,05	-	-	-	-	-
	PBD	0,28	0,30	0,07	-0,13	0,13	-0,28
Average Clustering *[2]	HC	0,25	-	-	-	-	-
	PBD	0,29	0,11	0,15	0,13	-0,07	-0,20
Characteristic Path Length *[2]	HC	-0,42	-	-	-	-	-
	PBD	-0,30	-0,32	-0,02	0,17	-0,09	0,26
Small World	HC	0,00	-	-	-	-	-
	PBD	-0,40	-0,35	-0,17	0,00	0,01	0,25
Global Efficiency *[1]	HC	0,37	-	-	-	-	-
	PBD	0,35	0,36	0,06	-0,07	0,09	-0,33
Local Efficiency *[1]	HC	0,29	-	-	-	-	-
	PBD	0,37	0,23	0,16	0,17	-0,13	-0,28
Modularity	HC	0,17	-	-	-	-	-
	PBD	-0,29	-0,17	0,05	0,32	-0,29	-0,16
Modules	HC	-0,25	-	-	-	-	-
	PBD	-0,17	0,19	0,29	0,49	-0,38	-0,33

*[1] Calculated on the normalised version of the SC matrices ([0,1]).

*[2] Weigted version; divided by random networks; uses the original SC matrices.

Supp. Table 5. Group differences in graph theoretical metrics.

Network metric	Controls		Bipolars		MW U-test	KS-test	t-test
	mean	SD	mean	SD	pvalue		
Mean Connectivity *[1]	0,119	0,005	0,120	0,007	0,619	0,589	0,818
Total Number of Fibers	422208,090	32796,155	412160,037	47764,226	0,534	0,890	0,507
Fibers per Connection	328,996	14,624	326,791	18,328	0,678	0,890	0,718
Degree	1284,667	99,853	1260,400	121,498	0,820	0,998	0,555
Connection density	0,160	0,012	0,157	0,015	0,820	0,998	0,555
Average Clustering *[2]	3,742	0,332	3,803	0,480	0,901	0,998	0,691
Characteristic Path Length *[2]	1,559	0,041	1,529	0,042	0,062	0,136	0,054
Small World	2,401	0,206	2,487	0,296	0,534	0,890	0,361
Global Efficiency *[1]	0,075	0,005	0,075	0,007	0,967	0,998	0,930
Local Efficiency *[1]	0,092	0,004	0,093	0,005	0,678	0,589	0,820
Modularity	0,596	0,012	0,594	0,016	0,740	0,589	0,735
Modules	7,133	0,516	7,267	0,458	-	-	-

*[1] Calculated using the normalised version of the SC matrices ([0,1]).

*[2] Weighted version; divided by random networks; uses the original SC matrices.

Supp. Table 6. Group differences in modular connectivity.

	Modular Network Analysis - Group comparison (HC Vs. PBD)				
Modules	degree	strength	pcoef	bwc	mod_deg_zscore
**1**	0,085	0,307	0,051	0,460	0,063
**2**	0,036	0,134	0,403	0,342	0,368
**3**	0,268	0,197	0,279	0,213	0,232
**4**	0,039	0,316	0,135	0,101	0,217
**5**	0,462	0,394	0,301	0,241	0,387
**6**	0,402	0,452	**0,006 ***	0,367	0,391
**7**	0,470	0,227	0,212	0,446	0,011

* survived FDR correction.
